# Gastro-oesophageal reflux - an important causative factor of severe tooth wear in Prader-Willi syndrome?

**DOI:** 10.1186/s13023-018-0809-3

**Published:** 2018-04-23

**Authors:** Ronnaug Saeves, Finn Strøm, Leiv Sandvik, Hilde Nordgarden

**Affiliations:** 1 TAKO-centre, Lovisenberg Diaconal Hospital, Pb 4970 Nydalen, 0440 Oslo, Norway; 2 Lovisenberg Diaconal Hospital, Oslo, Norway; 3 Department of Biostatistics and Epidemiology, Oslo University Hospital, Oslo, Norway

**Keywords:** GORD, tooth wear, Prader-Willi syndrome

## Abstract

**Background:**

Prader-Willi syndrome (PWS) is the most common genetic human obesity syndrome and is characterized by hypotonia, endocrine disturbances, hyperphagia, obesity and mild mental retardation. Oral abnormalities, such as decreased salivary flow rates and extreme tooth wear, have also been described. Studies have shown a significant increase in reflux symptoms in individuals with obstuctive sleep apnoea syndrome and increased BMI, both of which are typical findings in PWS. Gastro-oesophageal reflux disease (GORD) has been identified in some individuals with PWS and is a significant intrinsic factor in dental tooth wear. The aim of this study was therefore to estimate the prevalence of GORD in adults and children and to evaluate a possible correlation between GORD and tooth wear in adults with PWS. They were all registered at the TAKO-centre.

**Results:**

Twenty-nine individuals, 17 adults with a mean age of 32.6 years (range 18–48) and 12 children with a mean age of 8.8 years (range 3–17), agreed to undergo 24-hour oesophageal pH monitoring, and 90% of those enrolled managed to complete the examination. Four children and eleven adults were diagnosed with pathological gastro-oesophageal reflux, which is defined as acid exposure (pH less than 4) more than 3.6 or 4.3 percent of the time, respectively. Manometry performed in the adult group showed a pathologically high lower oesophageal sphincter pressure in four of the five individuals who had normal oesophageal pH values (pH under 4 less than 4.3% of the time). The two groups (reflux and non-reflux) were well balanced according to BMI, genotype, tooth grinding and hyposalivation. However, twice as many individuals in the reflux group as in the non-reflux group reported high consumption of acidic foods and drinks. Increased tooth wear was significantly correlated with GORD in the two groups (reflux *n*=6 and non-reflux *n*=6).

**Conclusions:**

The prevalence of gastro-oesophageal reflux is high in individuals with PWS. Tooth wear was strongly associated with GORD and acidic drinks, and both may be important aetiological factors underlying the extreme tooth wear in this group. Our data suggest a need for routine screening for GORD and dental wear in young individuals with Prader-Willi syndrome.

## Background

Prader-Willi syndrome (PWS) is a disorder affecting multiple organ systems and is the most common genetic human obesity syndrome. Epidemiological surveys estimate that its population prevalence reaches 1:52000 [1–3], and the gender ratio is close to 1/1 [1, 4]. The genetic mechanisms resulting in PWS are complex. The majority of individuals with PWS (70%) have a paternally derived deletion of 15q11-13, while maternal disomy 15 (UPD) occurs in 25% of individuals with PWS, and the remaining 2 to 5% have imprinting defects [5, 6]. The typical PWS deletion falls into one of two classes, type 1 or type 2, depending on the size and the chromosome breakpoint position. When the genotype-phenotype relationships become clearer, it may be clinically important to subtype the deletion classes [7]. Clinical diagnostic criteria have been developed, but as clinically overlapping disorders exist, the diagnosis must be confirmed by genetic testing [8].

PWS has a characteristic phenotype that includes severe neonatal hypotonia, early feeding problems, childhood onset hyperphagia, obesity, short stature associated with growth hormone deficiency, a high pain threshold and intellectual disability [9–11]. The syndrome has traditionally been described as having two nutritional stages: poor feeding and failure to thrive in infancy followed by hyperphagia leading to obesity in later childhood [11–13]. The aetiology of the switch from poor feeding to hyperphagia is thought to be associated with abnormalities in the hypothalamic circuitry [12]. The obesity can be controlled by strict dietary restrictions. Growth hormone treatment improves growth, physical phenotype and body composition [8]. The Necdin gene is important for the differentiation of central and peripheral sensory neurons and is congenitally absent in PWS [14]. A narrow forehead, almond-shaped eyes, down-turned corners of the mouth and a thin upper lip are characteristic facial features of PWS. Varying degrees of oral motor dysfunction are also common in affected individuals [15].

Thick, viscous saliva is a consistent finding in individuals with PWS [16–19]. Decreased salivary flow rates and increased amounts of salivary ions and proteins have also been reported [16, 17, 20] as well as severe tooth wear [18, 21, 22]. Tooth wear may result from attrition, abrasion, erosion or a combination of these factors. Attrition refers to the loss of enamel and dentin caused by the action of antagonistic teeth, while abrasion refers to the loss of tooth structure caused by other forms of physical wear due to mechanical processes involving foreign substances or objects. Erosion refers to chemical wear due to extrinsic or intrinsic acids [23, 24]. The microscopic structure of enamel and dentin has been found to be normal in teeth from individuals with PWS [25]. There are many causes of tooth wear, and it thus may be difficult to identify the aetiology in individual cases. However, the pattern of tooth wear in individuals with PWS suggests that erosive tooth wear is an important factor [22].

Gastro-oesophageal reflux disease (GORD) is an aspect of general health that may affect erosive tooth wear, and it has been described in one case report [26]. GORD is a significant intrinsic factor in erosive tooth wear [27–31]. Excessive daytime sleepiness, sleep apnoea and central obesity are common in individuals with PWS [32–34]. Studies have shown a significant increase in reflux symptoms in individuals with confirmed obstructive sleep apnoea syndrome and increased BMI (Body Mass Index) [35, 36]. Central adiposity may be the most important risk factor for the development of reflux [37]. To our knowledge no studies of gastro-oesophageal reflux in PWS have been published. Due to the extreme tooth wear in many individuals with PWS, GORD may be a serious problem and a causal factor in tooth wear for many individuals in this group.

The aim of this study was to explore the prevalence of pathological GORD in adults and children and to evaluate a possible correlation between GORD and tooth wear in adults with Prader-Willi syndrome. The null hypothesis was no difference in the prevalence of tooth wear between individuals with and without pathological gastro-oesophageal reflux.

## Methods

This study was performed at the TAKO-centre, a national resource centre for oral health in rare medical conditions (frequency fewer than 1:10 000), Lovisenberg Diaconal Hospital (LDH), Oslo, Norway. The study followed an observational cross-sectional study design.

The study protocol was approved by the Regional Committee for Medical Research Ethics and informed consent was obtained from all participants. For juvenile participants under 18 years of age and adult participants with guardians, informed consent was also obtained from a parent or guardian.

### Study participants

Fifty individuals, all of whom had been included in previous studies identifying salivary flow and tooth wear [19, 22], were invited to participate in the present study. They received written information, designed for both children and adults, describing the study. Eighteen adults responded and agreed to participate. One man who initially agreed to participate later changed his mind. Five children responded and agreed to participate. In addition, seven more children were included. All had been examined and followed at the TAKO-centre during the past three years, and the same data were available for them as for those included in previous studies. The final study group comprised 17 adults (11F, 6M, mean age = 32.6 years; range 18-48) and 12 children (6F, 6M, mean age = 8.8 years; range 3-17).

### Twenty-four-hour oesophageal pH monitoring

Seven adults were subjected to manometry and 24-hour oesophageal pH monitoring at Lovisenberg Diaconal Hospital, and six of them managed to complete the examinations, while ten individuals were evaluated at other local hospitals. Prior to and after each examination, the pH electrodes were calibrated using standard methods. Oesophageal manometry was performed in order to define the position of the sensor, 5 cm above the lower oesophageal sphincter (LOS), and the pressure of the LOS. Continuous pH recording was performed for 24 hours, and the total reflux time during the day and night was registered. The limits for pathological gastro-oesophageal reflux with acid exposure were set to a pH under 4 more than 4.3% of the time for adults [38] and 3.6% of the time for children [39].

Twelve children were referred to their local hospital to evaluate the degree of GORD. Ten of them underwent 24-hour oesophageal pH monitoring conducted at seven different hospitals throughout the country. None of the 29 participants had undergone any prior surgical treatment for GORD (i.e., open Nissen fundoplication), and none had been treated with percutaneous endoscopic gastrostomy (PEG).

### Clinical assessments

Children: BMI criteria for the age group 3-17 years of age (*n*=12) were age- and gender-adjusted by comparing their BMI with the age- and gender-specific cut-off values provided by the International Obesity Task Force (IOTF) [40]. Tooth wear was not evaluated in the younger age group (3-17 years) because many had mixed dentition. We also lacked baseline data from seven participants.

Adults: All adult study participants were examined once by the same examiner and underwent an anamnestic interview either during the consultation or, if parents or guardian did not attend the consultation, by telephone with a parent or guardian. The interview focused on oral and general health and nutrition, as well as symptoms of sleep disorders and gastric reflux. The frequency of consumption of acidic food and drinks was categorized as follows: more than once daily, once daily, several times per week, once per week, or never. Information about tooth grinding was also obtained.

BMI was calculated on the basis of measured height and weight. To define the BMI categories (kg/m^2^), the sample was divided into four groups (underweight (<19.9)), normal weight (20-24.9), overweight (25-29.9) and obese (≥30)).

Dental impressions (Aroma Fine Plus Normal Set, Alginate, GC Corporation, Tokyo, Japan) were collected for adult individuals. Tooth wear was evaluated using two indices, the Visual Erosion Dental Examination (VEDE) scoring system [41] and a modified individual tooth wear index (I_A_) [42]. The VEDE-index, a modification of the dental erosion index proposed by Lussi [43], is a 6-point scoring system that contains a visual guide with clinical photographs: 0=no erosive wear; 1=loss of enamel surface characteristics; 2=loss of enamel surface contour; 3=loss of dentine from less than one-third of the surface; 4=loss of dentine from more than one-third and less than two-thirds of the surface; 5=loss of dentine from more than two-thirds of the surface. An individual mean VEDE-score was calculated by summing up the surface score (labial/palatinal) evaluated by clinical examination and on dental casts for each tooth from upper right canine to upper left canine divided by the number of teeth present.

Tooth wear on the occluding surfaces was evaluated on dental casts and intraoral photographs using the I_A_ index. This index recorded tooth wear on a 4-point scale: 0=no or minimal wear; 1=wear of enamel down to dentine spots; 2=wear of the dentine down to one-third of the crown height; 3=wear of the dentine greater than one-third of crown height. In this study, the presence of a dental prosthetic crown due to tooth wear (according to the dental records) also qualified as a score of 3. The individual tooth wear index (I_A_) was calculated using the following formula: (10G_1_ + 30G_2_ + 100G_3_)/(G_0_ + G_1_ + G_2_ + G_3_), where G_0_, G_1_, G_2_ and G_3_ = number of teeth with occlusal wear scores of 0, 1, 2 and 3, respectively [42]. Tooth wear was first evaluated in all participants by four examiners in 2007/2008, and the results from that evaluation were originally described in a previous paper [22] and serve as a baseline for this report. In 2016, tooth wear was evaluated by one examiner (RS). The scores by RS in 2007 were close to the mean of the four examiners. The differences between the scores (I_A_ and VEDE) from 2007 and 2016 were used in the data analyses.

### Statistical analysis

When comparing tooth wear (I_A_- and VEDE index) between the two groups (the pathological gastro-oesophageal reflux and the non-pathological gastro-oesophageal reflux groups) an independent-samples t-test was applied. This application was based on the assumption that these variables are normally distributed. By using the findings from a relevant simulation study [44], we found that this assumption was adequately met for both variables.

A significance level of 5% was used throughout this work. The statistical analysis was carried out using the statistical software program (SPSS©; v. 24.0, SPSS Inc., Chicago, III., USA).

## Results

Anamnestic and medical information about the study group (n=29) is shown in Table 1. Three adults in the present study reported dysphagia and regurgitation. Four children and eleven adults were diagnosed with pathological gastro-oesophageal reflux with acid exposure (pH less than 4) more than 3.6 and 4.3 percent of the time, respectively (Table 2).

**Table 1:** Characteristics of the study population *n* = 29

	Age 3-17 yr n	Age 18-48 yr n	Total, n (%)
Sex			
Female	6	11	17 (59 %)
Male	6	6	12 (41 %)
Genetic mechanisms			
Del15	7	12	19 (66 %)
UPD15	5	5	10 (35 %)
Body mass index (BMI)			
Underweight (<19.9)	1	0	1 (4 %)
Normal weight (20-24.9)	8	4	12 (41 %)
Overweight (25-29.9)	1	5	6 (21 %)
Obese (≥30)	2	7	9 (31 %)
Sleep disorder			
Snoring	3	7	10 (35 %)
Enlarged adenoids	3	6	9 (31 %)
Apnoea /CPAP	3	6	9 (31 %)

Del15, deletion of the paternal chromosome 15q11-q13; UPD15, maternal uniparental disomy for chromosome 15; BMI was age- and sex adjusted for individuals 3-17 years according to the International Obesity Task Force (IONT) standards [40]. Sleep disorders were assessed by an ENT specialist

**Table 2:** Gastro-oesophageal reflux in the study population *n *= 29

	Age 3-17 yr n	Age 18-48 yr n	Total, *n* (%)
Gastro-oesophageal reflux			
Pathological	4	11	15 (52 %)
Non pathological	6	5	11 (38 %)
Reflux (clinical)	2		2 (7 %)
N/a		1	1 (4 %)

Two children did not complete the 24-hour oesophageal pH monitoring, but medication was initiated based on clinical signs.

The oesophageal position of the pH sensor, 5 cm above the lower oesophageal sphincter (LOS), was monitored. A pathologically high lower oesophageal sphincter pressure was reported in three of five individuals who had normal oesophageal pH values (a pH under 4 less than 4.3% of the time).

Data from the manometry and 24-hour oesophageal pH monitoring as well as the BMI, genotype, tooth grinding, intake of acidic foods and drinks and unstimulated whole saliva secretion for the 16 adults are presented in Table 3. One adult failed to complete the 24-hour oesophageal pH monitoring. Three individuals in the non-reflux group were evaluated and had been diagnosed with pathological reflux in 2007; they used reflux medication during the whole tooth wear registration period. The two groups (the reflux and non-reflux groups) were well balanced with respect to the following variables: BMI, genotype, tooth grinding and hyposalivation. However, twice as many individuals in the reflux group as in the non-reflux group reported high levels of consumption of acidic foods and drinks. The mean age was 26.8 years in the reflux group and 35.5 years in the non-reflux group.

**Table 3:** Twenty-four-hour oesophageal acid exposure and manometry findings in 16 adults

Individual	Age (yr)	Sex	Genotype	BMI	Reflux % Pat >4,3%^a^	LOS pressure (mmHg)^b^	Relaxation pressure (mmHg)^c^	UHS mL/min^d^	Tooth grinding	Acidic foods and drinks^e^
1	38	F	Del	24,2	29	30	Norm	0.17	no	1
2	26	M	Del	33,3	20	N/a	N/a	0.09	yes	1
3	20	F	UPD	41,1	4,8	N/a	N/a	0.05	no	1
4	42	M	Del	38,1	0,2	100	39	0.08	yes	1
5	41	F	Del	44,7	2,4	89	Norm	0.02	no	1
6	35	F	Del	37,6	2,9	84.9	Norm	0.33	no	0
7	25	M	Del	34,9	18	Norm	Norm	0.09	no	1
8	18	F	Del	27,6	6,9	N/a	N/a	0.04	yes	0
9	32	M	UPD	25,1	13,8	Norm	Norm	0.05	yes	1
10	40	F	UPD	23,0	0,8	91	Norm	0.24	yes	1
11	25	F	Del	26,4	9,6	16	Norm	0.16	yes	1
12	33	F	Del	22,5	0,3	N/a	N/a	0.06	no	0
13	34	M	Del	26,8	10,9	7.6	Norm	0.11	no	1
14	31	F	Del	31,0	27,1	44	29	0.2	yes	1
15	48	M	UPD	29	6	31	25.6	0.27	yes	1
16	31	F	Del	24,4	6,7	N/a	N/a	0.21	no	1

^a^Reflux (pathological > 4.3%)

^b^Lower Oesophageal Sphincter pressure (normal values: 13–43 mmHg)

^c^Relaxation pressure (normal value <15)

^d^Unstimulated whole saliva (hyposalivation ≤0.10mL/min)

^e^Acidic foods and drinks (sugared and diet soft drinks carbonated/not carbonated) more than once daily

Increases in tooth wear as the mean I_A_ and VEDE scores in the two groups (reflux *n*=6 and non-reflux *n*=6) registered over a mean of 7.5 years (3-9,5) are presented in Table 4. The increase in tooth wear was significantly correlated with GORD. Four individuals were excluded from the data analysis, two due to pathologically high LOS pressure (>90 mmHg) and two because they had dental crowns placed on all their teeth following the first tooth wear registration in 2007. Three individuals were diagnosed with GORD diagnosis and used proton pump inhibitors for the whole period of tooth wear registration; these individuals were placed in the non-reflux group.

**Table 4:** Tooth wear in the adult study population presented as mean I_A_ and mean VEDE

Indices	Pathological reflux *n* = 6	Non-pathological reflux *n* = 6	*p*-value
I_A_	14.63 ± 9.58	2.81 ±2.42	0.028
VEDE	0.65 ± 0.33	0.16 ± 0.14	0.013

Statistical significance (*p*<0.05)

## Discussion

As far as we know, this study is the first to investigate the prevalence of gastro-oesophageal reflux disease (GORD) using 24-hour oesophageal pH monitoring and to evaluate the association of GORD with tooth wear in a group of individuals with Prader-Willi syndrome. Fifty-two percent of the total study group (17 adults, 12 children) and 69 percent of the adults showed pathological gastro-oesophageal reflux. This study demonstrated a statistically significant association between GORD and tooth wear in the adult study group. The null hypothesis was therefore rejected.

The study group was small, and a larger group would have thus strengthened the results. However, PWS is a rare disorder, and as many as 17 of 26 adults who participated in the previous study in 2007 responded. Good cooperation was necessary to participate in the evaluation of 24-hour oesophageal pH monitoring, and when invited, several parents and guardians reported that it would be too challenging to participate in this study. The participants came from all over Norway, and for this reason, the 24-hour oesophageal pH monitoring needed to be completed at twelve different hospitals. Possible differences in the procedures may entail bias. Three individuals in the non-reflux group were evaluated and had been diagnosed with pathological reflux in 2007; they used reflux medication during the whole tooth wear registration period. Minor leakage to the oesophagus in this periode thus cannot be excluded.

The individuals were referred for their evaluation of their 24-hour oesophageal acid exposure, and data on the manometry findings were not always noted in the medical reports sent to us. However, pathologically increased lower oesophageal sphincter (LOS) pressure was reported in five of 16 individuals. Data on LOS was missing in five individuals, and those data would have strengthened the results.

All study participants were examined once by a single examiner (RS) and tooth wear was evaluated based on two indices using dental casts. In the previous study, four calibrated and blinded examiners evaluated tooth wear, based on the VEDE- and IA indices to counter potential bias. It was not possible to utilize four examiners during the last examination. However, the principal examiner (RS) was an average observer of the four, which indicates acceptable validity.

In this study, we were able to follow the development and increase in tooth wear for a mean of 7.5 years (3.0-9.5) for all adult participants. Moderate tooth wear may progress as a part of normal aging [45]. In the present study, the mean age of the reflux group was 27 years, and in the non-reflux group, it was 36 years. After adjustments for age and age-related physiological tooth wear, the difference in tooth wear between the two groups would have been even greater.

Gastro-oesophageal reflux is an aspect of general health that can affect erosive tooth wear [28–31]. Both the acid and fat contents of food are known to trigger GORD. In the present study, looking primarily at erosive tooth wear, we focused in particular on the content of acid and not fat in the diet. Individuals with PWS live on a strict diet (1100-1200 kcal) and eat regularely. Their diet is based on vegetables and light products, and the focus is on reducing the fat content of their food. We therefore, do not think that fat is a trigger for GORD in the participants. Focusing on the aetiology of tooth wear, all aspects of aetiology and consequences were not included in the study design. Studies have shown a significant increase in GORD symptoms in individuals with obstructive sleep apnoea syndrome, as well as increased BMI and central adiposity [36, 37] all of which are frequent risk factors and common to PWS [32]. The typical symptoms of GORD are heartburn and acid regurgitation. More atypical GORD symptoms may include a chronic cough, hoarseness, sleep disturbances and chest pains [46]. The prevalence of GORD varies in different parts of the world. The highest population prevalence has been reported in Europe (12–24%) [47, 48]. In a recently published Norwegian study [48], the prevalence of at least weekly GORD was found to be 17.1%, and that of severe GORD of 6.7% reported due to GORD symptoms. In our small group study, 11 of 16 (69%) adults underwent GORD diagnosis. Four out of five individuals in the non-reflux group presented with high lower oesophageal sphincter pressure >80 mmHg (normal range: 13-43 mmHg). The high LOS pressure may explain the minor leakage of acid to the oesophagus in these individuals. This condition can occur at any age, from early childhood to the ninth decade of life. The two participants who had LOS pressure of 90 and 100 mmHg (non-reflux) had received dental crowns on all their teeth due to extreme tooth wear after the first examination in 2007. For this reason, GORD at an earlier age cannot be excluded as an aetiological factor. None of the PWS study participants reported symptoms of reflux or heartburn in the anamnestic interview in 2007. The symptoms of GORD may have been underreported, possibly due to the high pain threshold of PWS, which could in turn lead to the decreased recognition of injury or illness [49]. Alternatively, individuals with the disorder may regard their long-standing reflux symptoms as “normal” and therefore not noteworthy. After starting on proton pump inhibitor medication, some of the participants reported GORD symptoms if the medication for some reason was stopped for a period. Three individuals in the present study reported dysphagia and regurgitation. This is in line with the findings of a study from 1987. Rumination is characterized by repetitive regurgitation of gastric contents into the oropharynx [50] and was reported by Alexander [51] in 53 out of 313 (17%) assessed individuals with PWS. No data have been published on rumination in PWS since 1987.

Tooth wear may be caused by attrition, erosion, abrasion or a combination of these processes. In the present study, two indices were used, one to focus on erosive tooth wear (VEDE) and one designed to measure abrasion or attrition on occluding surfaces (I_A_). Extensive tooth wear in PWS has been demonstrated and appears to be a significant problem [18, 22]. The multifactorial nature of tooth wear, influenced by variables such as hyposalivation, tooth grinding, and intrinsic (gastric) and extrinsic acids (most commonly dietary), makes it difficult to establish its aetiology. GORD is a significant intrinsic factor in erosive tooth wear [29, 30] and is consistent with our obsevation of a strong association between GORD and tooth wear. The microscopic structure of enamel and dentin in teeth from individuals with PWS has been found to be normal. The enamel surface was generally smooth without structure, but could in some aspects resemble the effects of an acidic agent [25].

Earlier studies found that saliva protects the teeth against tooth wear [21, 52]. In our previous study, the low salivary flow rate did not maintain a significant association with tooth wear and is therefore possibly only a minor contributory factor in extreme tooth wear. In the present study, hyposalivation was well balanced in both the reflux and non-reflux groups. Dietary acids are considered to be the most common cause of erosive tooth wear by many researchers [30, 53, 54]. These results are in accordance with the findings of the present study.

## Conclusion

To our knowledge, this is the first study addressing GORD in Prader-Willi syndrome. The prevalence of GORD seems to be high and to increase with age in PWS. Tooth wear was strongly associated with GORD and the intake of acidic drinks, and both may be important aetiological factors for the extreme tooth wear observed in this group. It is important to be aware that GORD may be a health challenge in individuals with PWS, and more studies on this are clearly necessary. Our data suggest a need for routine screening for GORD and tooth wear in young individuals with Prader-Willi syndrome.

## Abbreviations

BMI: Body mass index
GORD: Gastro-oesophageal reflux disease
I_A_: Individual tooth wear index
LOS: Lower oesophageal sphincter
PWS: Prader-Willi syndrome
VEDE: Visual erosion dental examination
